# Real-Time Telemetry System for Monitoring Motion of Ships Based on Inertial Sensors

**DOI:** 10.3390/s17050948

**Published:** 2017-04-25

**Authors:** José M. Núñez, Marta G. Araújo, I. García-Tuñón

**Affiliations:** 1 Centro Universitario de la Defensa (CUD), Escuela Naval Militar (ENM), Plaza de España s/n, 36920 Marín, Pontevedra, Spain; 2 Departamento de Teoría de la Señal y Comunicaciones, Universidad de Vigo, Campus Universitario Lagoas-Marcosende, 36310 Vigo, Pontevedra, Spain; martaga@com.uvigo.es (M.G.A.); inesgt@com.uvigo.es (I.G.-T.)

**Keywords:** Inertial Measurement System (IMU), Inverse Synthetic Aperture Radar (ISAR), radar imaging, telemetry, radio data link

## Abstract

A telemetry system for real-time monitoring of the motions, position, speed and course of a ship at sea is presented in this work. The system, conceived as a subsystem of a radar cross-section measurement unit, could also be used in other applications as ships dynamics characterization, on-board cranes, antenna stabilizers, etc. This system was designed to be stand-alone, reliable, easy to deploy, low-cost and free of requirements related to stabilization procedures. In order to achieve such a unique combination of functionalities, we have developed a telemetry system based on redundant inertial and magnetic sensors and GPS (Global Positioning System) measurements. It provides a proper data storage and also has real-time radio data transmission capabilities to an on-shore station. The output of the system can be used either for on-line or off-line processing. Additionally, the system uses dual technologies and COTS (Commercial Off-The-Shelf) components. Motion-positioning measurements and radio data link tests were successfully carried out in several ships of the Spanish Navy, proving the compliance with the design targets and validating our telemetry system.

## 1. Introduction

Knowledge and reduction of the Radar Cross-Section (RCS) or radar reflectivity of warships is essential in a modern navy. Determination of the units’ RCS is vital to minimize the possibility of the detection of the ship itself and to get an efficient employment of active countermeasures. For precision RCS measurements, High Resolution Radar (HRR) techniques as ISAR (Inverse Synthetic Aperture Radar) [1] can be used. ISAR allows obtaining an electromagnetic image of the target from which it is possible to detect hot spots and, in some cases, identify the target. However, rotational and translational motions of the ship relative to the ISAR radar system affect the quality of the image. A small degree of rotational motion is required in order to generate Doppler shift necessary to form the image, but on the other hand, the remaining motions of the target (translational motion and large degree rotational motions) degrade ISAR image quality. Translational motion leads the target scatterers to migrate to other range bins during the illumination time [2]. Also during this time, large rotational motions will cause more distant scatterers, migrate to neighboring Doppler cells degrading the final image. These movements must be compensated to prevent the image from becoming blurred or distorted, which would make the details hardly recognizable, complicating subsequent analysis, recognition and classification processes [3,4].

Numerous techniques try to mitigate this problem by a posteriori algorithms that compensate translational and rotational movements of the ship [5], but many of these techniques need to estimate the dynamics of the ship to be effective. When the target is collaborative, such as its own unit, it is crucial to have a system that records the accurate response of the ship (yaw, pitch and roll) and position information of the unit during the measurement evolution, for later use in correcting and focusing the generated ISAR images.

**Figure 1:**
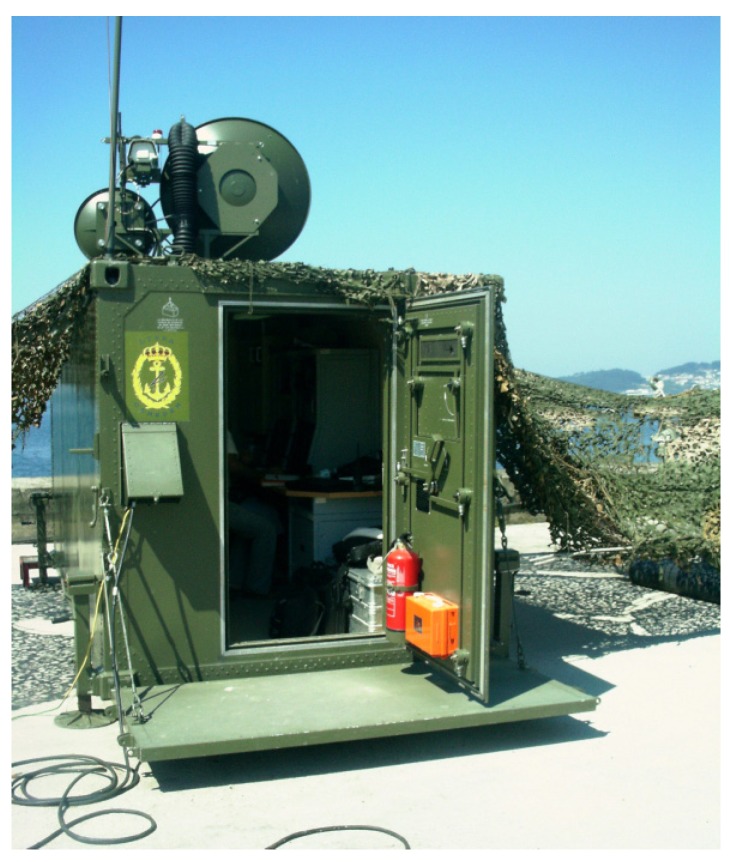
LIBRARCS measurement system.

The necessities of the HRR-imaging field pointed out above gave rise to this work. Let us give some background to the work. In 2001, by request of the Spanish Navy, the company INDRA and the Antenna Group of the University of Vigo undertook the development of a moderate cost measurement system of mean RCS referred to as LIBRA [6]. The system has characterized until now almost all radar signatures (RCS) of the Spanish Navy warships. It is portable and housed in a container, which is moved to the location of the measurement campaign (Figure 1).

**Figure 2:**
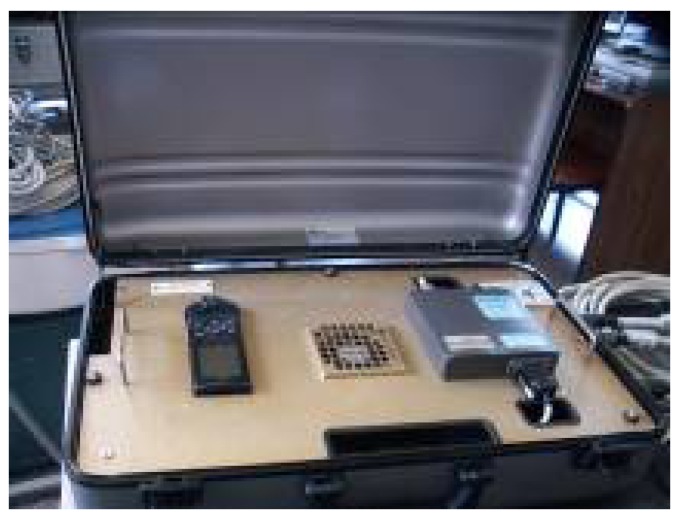
LIBRA remote unit (URL).

LIBRA is made of several subsystems. One is the target tracking subsystem that has a remote unit (URL), which is installed on-board to transmit real-time position, behavior and navigation data of the ship (Figure 2). Positioning information is obtained from a GPS (left on the figure), and the response of the ship is logged from a side by side fiber-optic gyrocompass. All data are transmitted to the on-shore system using a low speed radio modem.

The LIBRA system does not meet the needs nowadays mainly due to the following drawbacks related to the URL:

1. It needs a high deployment time.
2. The laser gyro is very heavy and must be stabilized for hours prior to measurements. Furthermore, the cost of this unit is very high.
3. The radio data link is unstable and too slow for the requirements demanded by the high resolution radar imaging process.
4. An external power supply is required, so it is not completely autonomous.
5. It is a custom design, so parts and maintenance have a great economic cost, unacceptable in most cases.
6. The system does not incorporate any redundancy mechanism, so that in extended measurement campaigns, a failure in one sensor would prevent taking measurements, forcing aborting of the campaign.

The lacks detected in the URL of LIBRA demanded a study of feasible alternatives to replace the URL looking for new hardware and software designs.

There are very few RCS measurement systems for warships. As an example, we will mention the NADIRsystem of the company DS Ingegneria Dei Sistemi S.p.A. [7], the RCS measurement system from SISPAS [8] and the instrumental radar “BlueMax G6” developed by Star Dynamics Corporation [9]. In addition, there is a great opacity in accessing the technical details of the implementation of this type of system, as corresponds to measurement equipment for the estimation of parameters related to the defense and surveillance of warships. Scientific communications are very rare, except those with very general information.

Due to the opacity mentioned above in this field of research, our working group faced a pioneering work with the only previous experience of the LIBRA system developed years before for the Spanish Navy. As a result, a low-cost system for measurement, recording and transmission of real-time data of pose estimation and the position of ships at sea has been designed, and it is described throughout this paper.

This paper is organized as follows: Section 2 provides an overview of the system architecture; Section 3 describes the hardware and software implementation of the measurement node; Section 4 analyses the redundancy of the system; Section 5 presents the Over The Air Programming (OTAP) procedure; Section 6 shows the results of the test campaigns; and finally, Section 7 provides the conclusions.

## 2. Overview of the System Architecture

**Figure 3:**
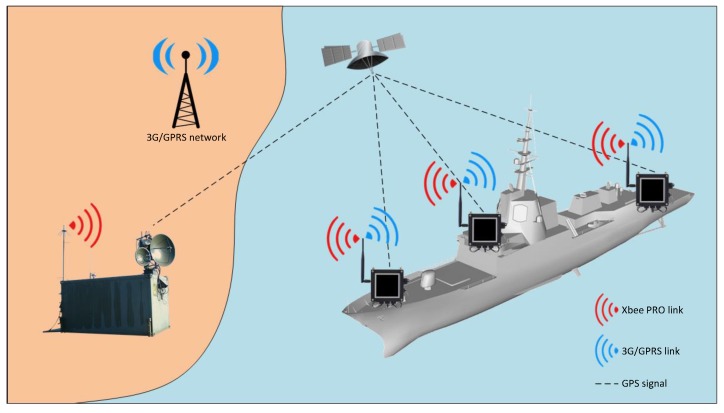
System operation overview.

It is shown in Figure 3 that the proposed system is divided into two segments or parts. The first one is the on-shore or ground installation, and the second one is the on-board equipment.

The on-board part consists of one or more units (nodes) arranged in different parts of the ship. These units collect, store and transmit behavior and position data of the vessel in real time. Moreover, the ground installation merges data from the nodes to deliver unique information to different data consumers. Its composition and working details are detailed in Section 3.

**Figure 4:**
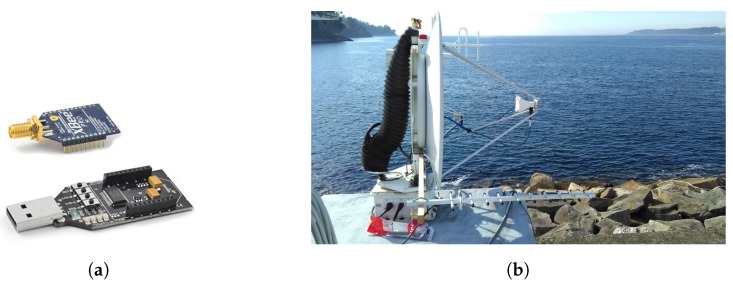
Ground station: (**a**) Waspmote Gateway XBee radio module; (**b**) Yagi antenna.

The ground unit is based on a Waspmote Gateway [10] with a XBee radio module (Figure 4a) attached to a laptop USB port and connected to an 11-element Yagi antenna (Figure 4b). The gateway, once connected to the PC, creates a new serial port on the computer that connects any application with the XBee module allowing receive or send data packets to the network measurement nodes and performs OTAP functions (see Section 5). The ground equipment is completed with a 3G-GPRS modem, which supports the secondary or backup link.

## 3. Measurement Nodes

**Figure 5:**
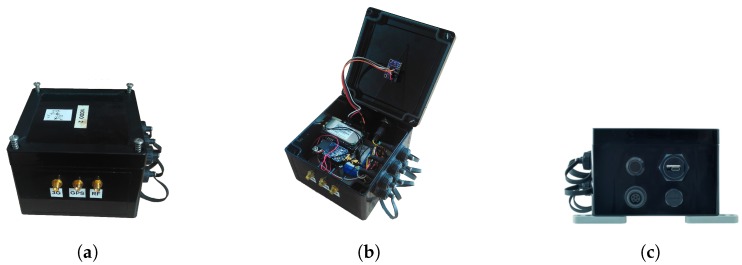
Measurement node: (**a**) enclosure with RF connectors; (**b**) electronics and IMU (under the lid); (**c**) switch button and USB connector.

The measurement node is enclosed in an IP-65 waterproofed ABScase as shown in Figure 5a, where one can see also the GPS and radio module connectors. This enclosure warranties a perfect seal that protects against inclement weather and sea water. Figure 5b exhibits the electronics, housed inside the case, and the IMU, which is firmly joined to the lid. The module also has a power switch with an LED indicator and a USB connector for local programming and battery charge (Figure 5c).

**Figure 6:**
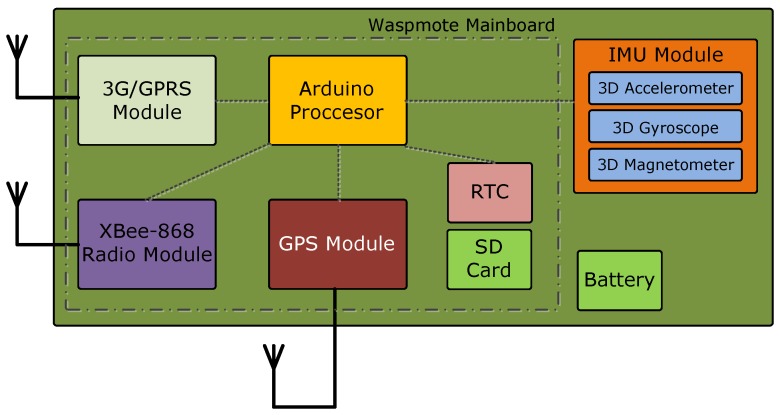
Measurement node composition.

Figure 6 illustrates the measurement node composition. The main board contains an Arduino-based microcontroller and peripherals and also holds the RF modules. The XBee-PRO-868 transceiver and the 3G-GPRS radio modem give the node communication capability by establishing a primary XBee radio link with the ground station and a 3G-GPRS backup link.

GPS and IMU comprise the system sensors. The GPS module allows the estimation of the node position, course and speed of the ship. Meanwhile, the IMU provides pose data (pitch, roll and yaw).

The node has a Real-Time Clock (RTC) that, in case of the unavailability of the GPS signal, would also mark frames with a time stamp. When a new measurement is started, the RTC is initialized with the GPS time that can be captured with a single satellite in view. Finally, a battery is installed inside the node, which can also be powered from the USB connector.

### 3.1. Main Board

**Figure 7:**
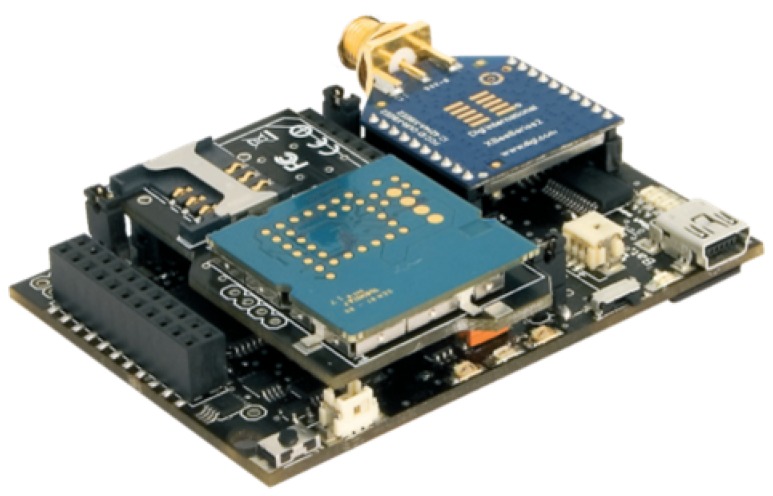
Waspmote node main board with attached XBee module.

The main board is an Arduino-based open source wireless sensor platform called Waspmote [10]. Its modular design philosophy allows an XBee-PRO-868 [11] radio module, a 3G-GPRS radio modem and a GPS receiver to be connected to the main board, generating a compact and robust design, as shown in Figure 7. The different modules are powered by the main board, which also provides various data buses for peripherals’ communication, analogs and digital inputs/outputs and two UART (Universal Asynchronous Receiver-Transmitter).

### 3.2. Main Radio Link

Primary radio link is based on XBee-PRO-868 radio modules. These transceivers work on the 868-MHz ISM radio band. The ISM bands are internationally reserved parts of the Radio Frequency (RF) spectrum, intended for Industrial, Scientific and Medical requirements rather than for communications. ISM bands are also called unlicensed bands, which vary according to different regions. USA and EU regulatory agencies place limitations for these ISM bands on the operating frequencies, output power, spurious emissions, modulation methods and transmit duty cycles, among other things. One of the most used ISM bands in Europe is centered at 868 MHz. Due to those regulations, transmitted power of XBee-PRO-868 modules is limited to 315 mW with a maximum RF data rate of 24 kbps. This regulatory restriction constrains the operational range of the system as described in Section 6.3.

### 3.3. Spare Radio Link

**Figure 8:**
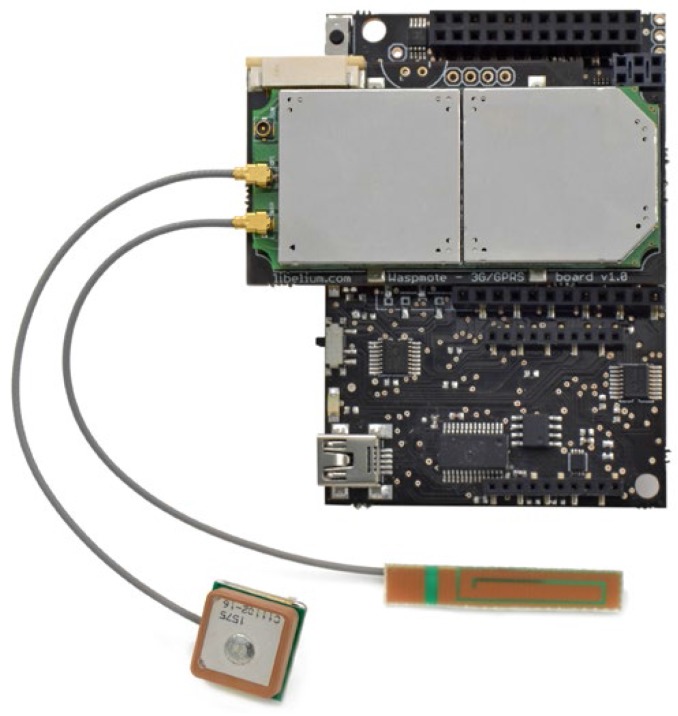
SIM5218E. 3G-GPRS module for the spare radio-link.

The 3G-GPRS module, an SIM5218E from SIMCom [12] (Figure 8), is used to establish the secondary or backup link. The modem is compatible with 3G, WCDMA, HSPA, UMTS, GPRS and GSM networks and employs an external antenna attached to one node outside connector (Figure 5a).

The secondary or backup link is activated when the unavailability of the main link is detected for 10 s (this time is configurable). The module has no power until that moment, so that the node does not consume battery if not used. Once the module is powered, it takes about 60 s to register on a 3G network.

The module can act as a TCP/IP server, but the preferred configuration is only one server running in the ground station against placing several servers, one on each measurement node. On the other hand, with a single server located on-shore, the measurement node software is simplified, which has a limited processing capability, and data are ensured to be centralized.

The method chosen to transfer telemetry frames is based on the HTTP protocol connections because it is standard, simple and requires little computational load. On the ground station, the PC equipped with a 3G radio modem runs an Apache Web server. Measurement nodes access simultaneously this server by sending GET commands with the following format:

http://<host>/parser.php?<query>, where <query> represents telemetry data being transmitted fitting the format:

<variable1=value1>&<variable2=value2>&...

For example, http://myhost.es/parser.php?<TR=7336>&<BL=81>&<TP=15>&<DC=5> indicates the server for which at instant 7336 ms, there is a battery level of 81% and the expended RF duty cycle is 5%.

One of the most interesting features of this module is that it enables the implementation of the OTAP gateway feature, which allows remotely upgrading the firmware of measurement nodes as explained in Section 5.

### 3.4. Inertial Measurement Unit

**Figure 9:**
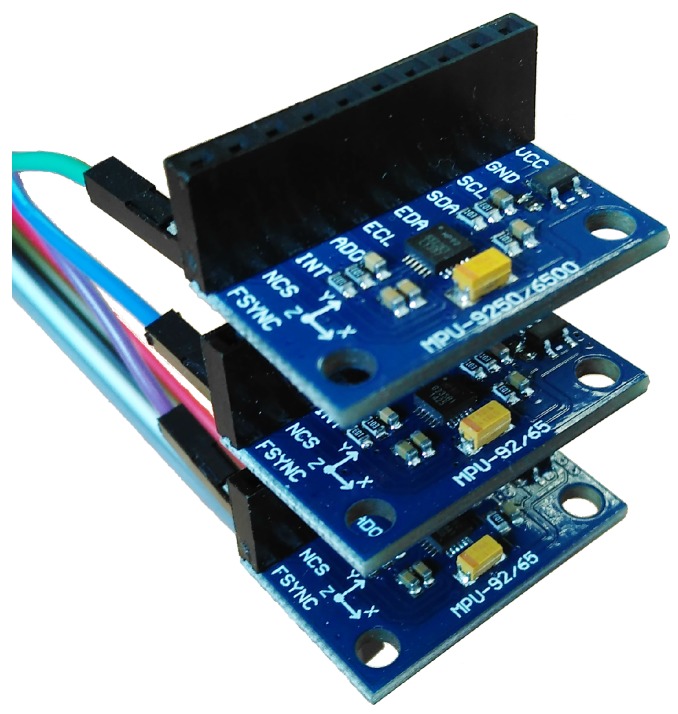
Inertial sensors block.

The inertial measurement unit block consists of three equal and independent MPU-9250 [13] IMU modules, as shown in Figure 9. Each module is composed of one MPU-6500 inertial sensor and one AK-8963 three-axial magnetometer connected through an internal I2C. Three axial accelerometer and a gyroscope form the inertial sensor. The main characteristics of the MPU-9250 can be found in Table 1.

**Figure 10:**
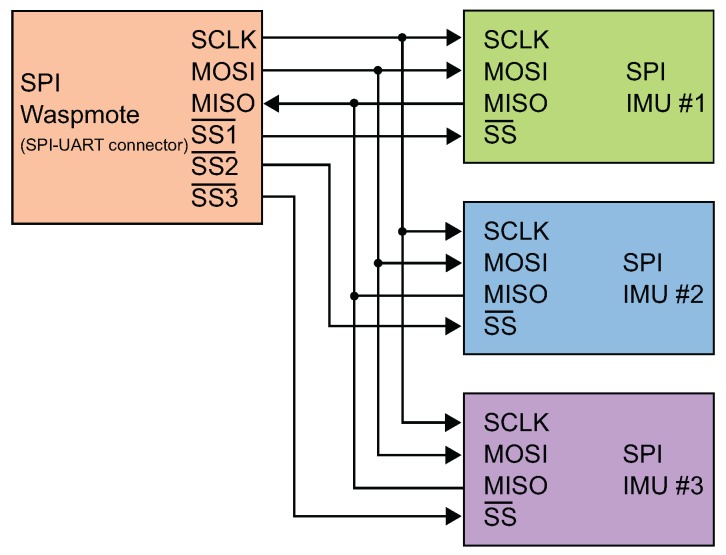
SPI bus implementation to interconnect the inertial sensors block and the main board.

The MPU-9250 modules are connected together by an SPI bus with the Waspmote main board as indicated in Figure 10. The developed software is responsible for selecting each module using the SS (Slave Select) digital output line. The SS line is normally held high, which disconnects the module from the SPI bus, so to talk to a particular module, the corresponding SS line should be lowered, and the rest of them should be kept high. When SS is low, an individual MPU-9250 is selected, and it can begin to transmit data through the MISO (Master In Slave Out) line or receive data through the MOSI (Master Out Slave In) line. The Serial CLocK (SCLK) line generates the clock signal.

With this procedure, individual data from the accelerometer, gyroscope and magnetometer of each MPU-9250 module can be requested. Once inertial sensors’ data are obtained, ship response data are obtained by means of a fusion filter.

Redundant sensors can provide highly accurate sensor data and also reconfigure sensor network systems if some sensors fail. Through increased redundancy, we can obtain noise reduction in the ship response output parameters. The inertial measurement unit block in Figure 9 is based on a redundant linear IMU configuration where several accelerometers and gyro sensors are mounted on each block axis. The three independent IMU modules composing the block work on a redundant configuration to improve the measurement quality acting as a “synthetic” or “virtual” IMU.

The idea of combining measurements from multiple sensors into a single, higher-accuracy virtual IMU, was first proposed in [14], where a Kalman filter was employed to combine sensor readings, which is optimal under the assumption of a linear system with additive Gaussian white noise.

As is stated in [15], from a theoretical point of view, the best estimate of the expected value $\hat{x}$ of *n* independent measurements $x_{1},...,x_{n}$ (with their respective variances $\sigma_{1}^{2},...,\sigma_{n}^{2}$) can be computed as a weighted average of the measurements:

(1) $$\hat{x}=w_{1}\cdot x_{1}+w_{2}\cdot x_{3}+\cdots +w_{n}\cdot x_{n}=\sum_{i=1}^{n}w_{i}\cdot x_{i}$$

where $w_{i}$ are the weighting factors. Assuming homogeneous measurements (i.e., constant $\sigma_{i}$), its variance $\sigma_{\hat{x}}^{2}$ can be derived as:

(2) $$\sigma_{\hat{x}}^{2}=\sum_{i=1}^{n}w_{i}^{2}\sigma_{i}^{2}$$

If all sensors have the same measurement variance, $\sigma_{x}=\sigma_{i},\;\forall i$ and the weighting factors are all equal, then the variance results:

(3) $$\sigma_{\hat{x}}^{2}=\frac{\sigma_{x}^{2}}{n}$$

**Figure 11:**
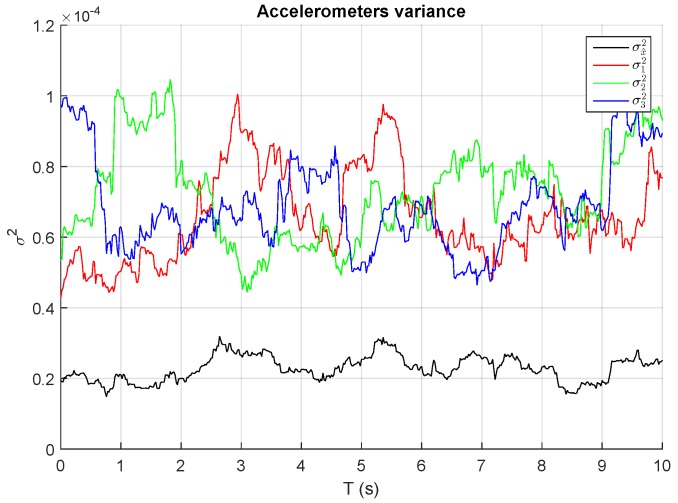
Individual measurement variances and sum block variances of three accelerometers in steady state with equal weighting factors.

As is indicated by the above equation, the variance of the output noise of a block of *n* sensors is inversely proportional to the number of sensors, i.e., the variance of the block output noise is *n* times lower than the individual sensor variances. Figure 11 depicts the variances of n=3 different accelerometers measurements in steady state and the total variance of the sum assuming equal weights. As expected, total variance or noise is reduced approximately by three times, confirming the validity of the model.

Some data fusion algorithms use constant weighting values estimated through prior measurements realized for each sensor. Nevertheless, as suggested in [16], if the variance of one sensor is significantly different from the variance of the others, a method for adapting the weights $w_{i}$ to each sensor variance is required. In this method, each sensor should have a weight that is inversely proportional to its standard deviation.

**Figure 12:**
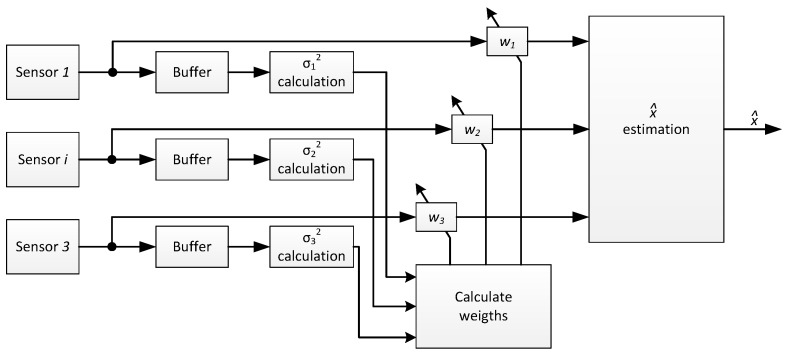
Proposed weighting algorithm for $\hat{x}$ estimates using three sensors.

A step further consists of adapting continuously the weights $w_{i}$ to the best estimate calculus based on the variance of the independent measures given by their $\sigma_{i}$. Different methods have been proposed to best adapt weighting factors [16,17]. The method proposed in [17] is implemented in the measurement nodes of this work. In the proposed algorithm, the weighting factors of each sensor $w_{i}$ are adapted in real time as a function of the instant variance of each sensor, as is illustrated in Figure 12.

**Figure 13:**
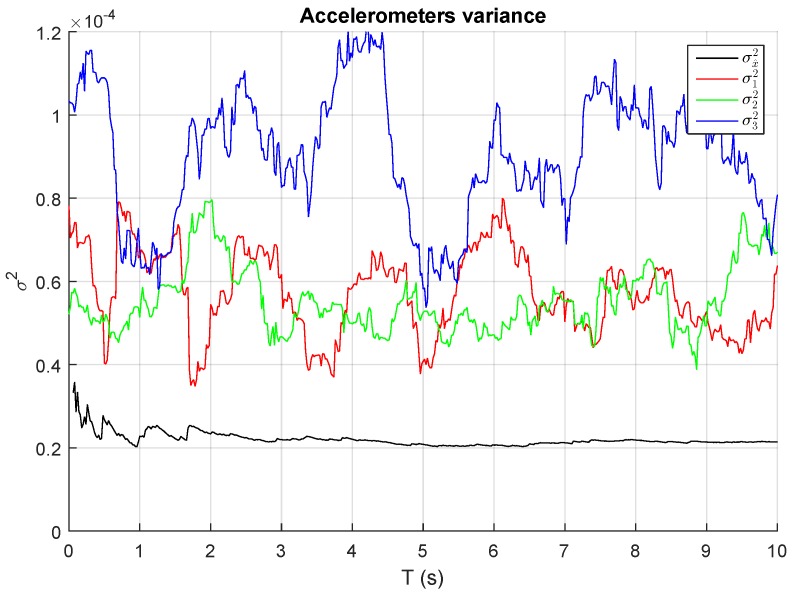
Individual measurement variances and sum block variances of the three accelerometers in steady state with adaptive weighting factors.

**Figure 14:**
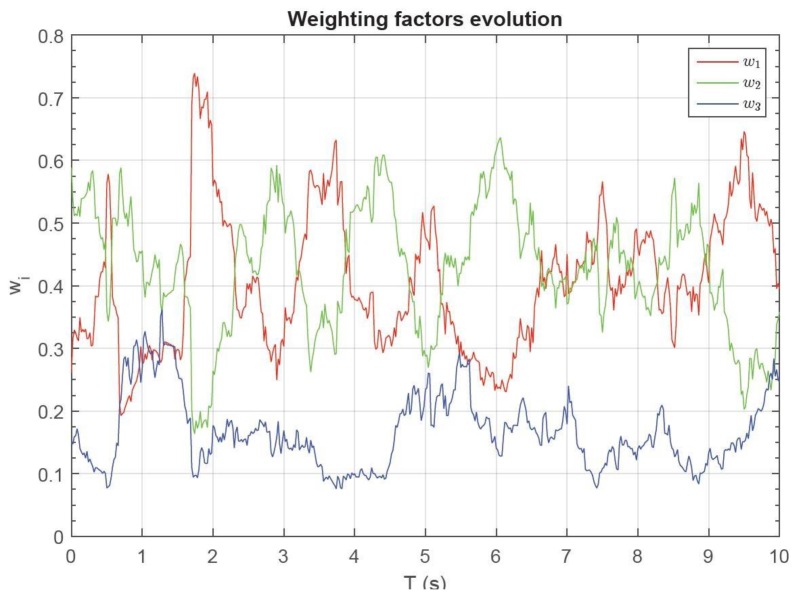
Evolution of the adaptive weighting factors.

As can be seen in Figure 13, by means of adaptive weighting factors, additional noise reduction in the sensor output is achieved. The evolution of weighting factors versus time is plotted in Figure 14.

#### 3.4.1. Sensors Calibration

The three MPU-9250 devices (InvenSense, San Jose, CA, USA) conforming the IMU block are composed by MEMS (Micro Electro-Mechanical Systems) accelerometers, gyros and magnetometers. The performance of this type of devices is degraded by fabrication defects, asymmetries in the produced structures, misalignment of actuation mechanisms, deviations of the center of mass from the geometric center and thermal stress formed in the solder of the sensor during the reflow soldering process, IMU package misalignment errors and IMU sensor-to-sensor misalignment errors. Therefore, a procedure to calibrate the sensors effectively is required to reduce the errors and to increase the IMU block precision.

**Figure 15:**
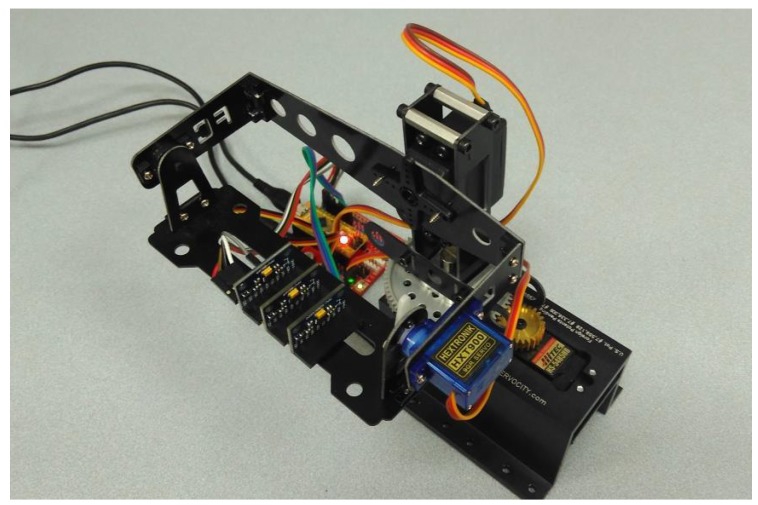
Three DoF positioner with the three IMU blocks mounted on it.

The calibration procedure proposed in [18] is used by simplicity, which includes a calibration scheme and a calibration algorithm. The procedure consists of putting the IMU block on its six standard positions. In each of the six positions, the accelerometer, gyro and magnetometer output will be measured for a few seconds and then averaged to reduce the measurements noise. This procedure is automatically performed, as shown in Figure 15, using a three degrees of freedom (DoF) positioner.

The required calculations are effortlessly implemented in the sensor nodes with a very low computational cost. The main advantage of this method is that it requires only simple measurements that can be easily performed by the user in a few minutes without any extra tools. Moreover, the method is still valid in the non-stationary case.

#### 3.4.2. IMU Data Fusion

**Figure 16:**
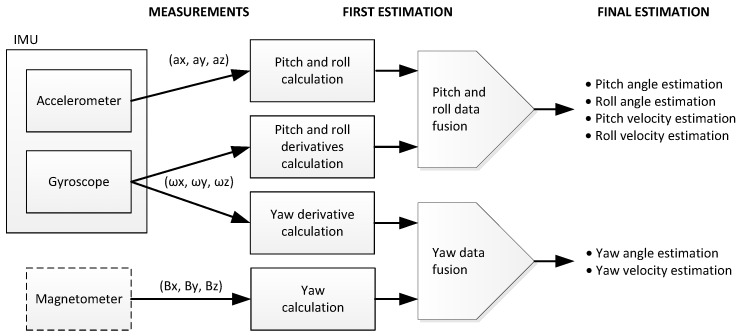
Data fusion between accelerometer, gyro and magnetometer to estimate roll, pitch and yaw.

From the measurements obtained from any of the three on-node sensors (accelerometer, gyroscope and magnetometer), it is theoretically possible to estimate the vessel motion data (Figure 16), although the ideal solution is to combine all three. The fused redundant attitude reduces uncertainty, increases accuracy and reliability in the case of sensor failure.

As shown in Figure 16, pitch and roll estimation can be obtained from accelerometer and gyroscope data fusion, and the yaw angle is estimated from magnetometer and gyro data. Fusion is implemented using different algorithms or fusion filters. A large variety of filters can be implemented in the nodes for data sensor fusion, like the one proposed by Mahony [19], Madgwick [20], or even any variant of the Kalman filter [21]; however, in this case, the complementary filter of [22] is selected by simplicity, giving excellent results.

**Figure 17:**
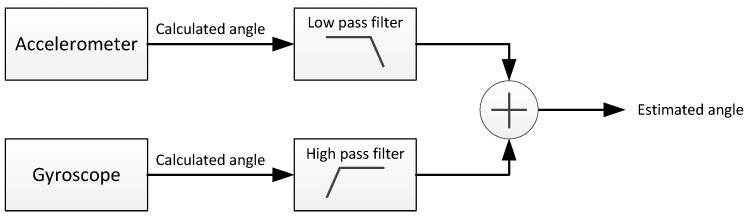
Scheme of the data fusion process based on a complementary filter.

As depicted in Figure 17, by passing the accelerometer data through a low-pass filter, high-frequency perturbations are eliminated, and the accuracy of the long-term results is exploited. Analogously, when the gyroscope signal is filtered with a high-pass filter, the negative effects of the bias are eliminated, and the benefits of the long-term estimations are taken as an advantage. The working principle of this filter responds to the following equation:

(4) $$\theta_{k}=\underset{\mathrm{high}-\mathrm{pass}\;\mathrm{filter}}{\underset{︸}{\alpha \cdot \underset{\mathrm{angle}\;\mathrm{integration}}{\underset{︸}{\left(\theta_{k-1}+\omega_{k}\Delta t\right)}}}}+\underset{\mathrm{low}-\mathrm{pass}\;\mathrm{filter}}{\underset{︸}{\left(1-\alpha \right)\cdot a_{k}}}$$

where $a_{k}$ represents the measured acceleration value in the *k* instant, $\omega_{k}$ is the measured angular velocity, $\theta_{k}$ represents the estimated angle, Δt is the sample time and α would actually represent the weight of each filter (low-pass and high-pass) in the global filter. This filter is implemented by software with this simple algorithm:

(5) $$\theta_{k}=\alpha \theta_{k-1}+\left(1-\alpha \right)a_{k}+\alpha \omega_{k}\Delta t$$

### 3.5. GPS

A GPS is installed inside the measurement node to obtain the geographical position and the course of the vessel relative to stationary land or COG (Course Over Ground). GPS module selected is JN3-Jupiter from Telit [23]. The module is attached to the main board through one of the UART and also connected to an external active 28 dB gain antenna.

**Figure 18:**
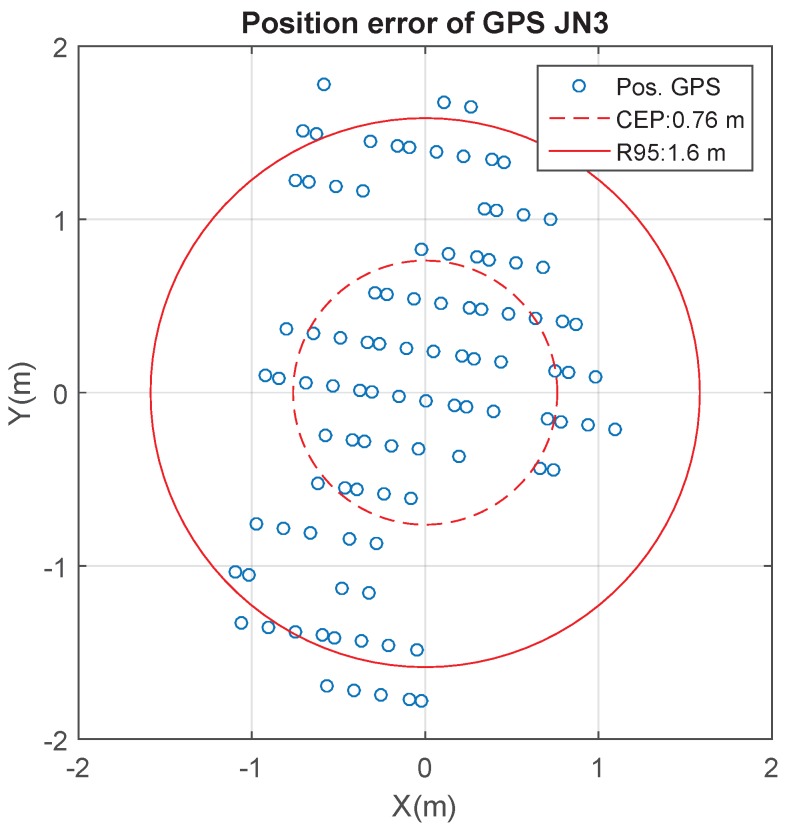
Static position errors on the *x* and *y* axes of the JN3 GPS.

Position errors for this GPS module have been characterized resulting a CEP (Circular Error Probable) of 0.76 meters and a 95% accuracy (R95) of 1.6 m, as is shown in Figure 18.

In addition to providing the vessel positioning and COG, this module has another important role: providing the GPS time reference. All frames are marked with GPS universal time, internally stored and transmitted, so that they can be aligned with the data of the radar system, since it uses this same time.

### 3.6. Data Storage

The motion data, position, speed and course of the vessel and the time information in which they were acquired are stored in each node on a microSD memory card, so in a supposed situation that prevented the node from transmitting, the node data could be extracted and integrated later, provided that the ISAR image processing is performed off-line. The measurement files are limited to 5 min of measurement duration (although this parameter is variable). Reaching this time, a new file is created. Transmitted frames are identical to those that are stored. Such frames have a variable length and are formed by several field identifiers followed by values. As an example, a transmitted frame may be:

$TR:995693$AX1:0.23$AY1:0.32$AZ1:−1.68$BL:88$TP:24

which means that, at a relative time of 995,654 ms, the triad value of the first accelerometer is $\left\{0.23,0.32,-1.68\right\}$, the battery level is 88% and the expended RF duty cycle is 24%.

### 3.7. Power Supplying System

The node is powered with an internal Li-ion battery, which is able to provide 3.7 V and 6600 mA/h. Components and modules connected to the main board can be switched on and off depending on whether its use is necessary or not, to limit battery consumption of the node. The main board includes a charge controller that manages the battery charging process using different sources (USB port and solar panel) and monitors the battery state.

Total consumption of a node, when the RF and GPS module are working simultaneously, is 560 mA [11,23], which would give the node a theoretical autonomy of 10 h. However, according to the tests carried out and taking into account the degradation of the battery due to the number of load cycles, the real average autonomy is about 8 h. The main consumer in the node is XBee module, which demands 90% of the total power, as indicated in Table 2.

In the case that the radio link 3G-GPRS support is used, the total consumption of Table 2 would be reduced to 377 mA (assuming a sustained data transmission in GSM mode to full power), so autonomy will be improved by 5 h.

The measurement node can be powered also from an external source via a USB port to charge the battery. In this case, the current is limited by the charge controller to 100 mA, which slightly increases the autonomy of the node.

## 4. Redundancy

The high-resolution radar measurement campaigns can last for several hours, forcing two key factors to be considered in the design of the measurement nodes: first, the energy independence of the nodes and, second, the high degree of reliability required. Indeed, the daily cost of sailing a warship is very high, so nodes must be designed to include elements of redundancy, ensuring data availability in any scenario. The developed system presented in this work has several strengths that guarantee the data availability. They are summarized below:

- The availability of several nodes measuring attitude data, position, speed and course of the vessel (which are practically the same for all nodes) can not only improve the accuracy of such data, but also ensure that the system continues to operate if one or more nodes cannot transmit or fails.
- As exposed in Section 3.3, if the XBee-PRO-based radio link is not available, either by interference, lack of range or transmitter failure, a 3G/GPRS module automatically assumes the transmission of frames to the on-shore station. If this situation occurs, the system range is restricted to the mobile network coverage in the measurement zone.
- The storage of the data of each node in a microSD makes possible the data recovery if it is needed.
- The internal RTC of each node provides the time stamp to the data frames if the GPS signal is unavailable.
- The attitude of the target can be determined from the data supplied by the node internal IMU and also by the gyroscope of the ship, through one of the auxiliary UARTs. As discussed in Section 3.4, the IMU information is redundant, because it comes from the data fusion of a gyroscope, an accelerometer and a magnetometer.
- As indicated in Section 3.7, each node is autonomously supplied by an internal battery, which give more than 8 h of autonomy. This time can be significantly extended when the node is externally supplied through a USB port.

## 5. Over The Air Programming

OTAP, a concept initially bound to the remote updating of smartphones, is taking a new direction with the appearance of WSNs and the Internet of Things. After the deployment of a WSN, the possibility of reprogramming the nodes remotely becomes essential to deal efficiently with the required maintenance and updating tasks. In our case, this is particularly useful, as boarded nodes can be reprogrammed remotely from the ground without having to access the ship on sea.

The OTAP procedure is very attractive not only from the point of view of the testing functionality, but also to modify algorithms or procedures on the fly and change the operating parameters of the IMU, GPS or 3G/GPRS module. OTAP allows modifying the node functionality without having to physically access it, by loading a new program or a version stored in the microSD card.

The OTAP functionality can be performed with the primary radio link (XBee radio module) or with the secondary link (3G-GPRS module). With the first one, software updates could be made up to approximately 8 km, which is the maximum effective distance of the radio link. In the case of using the secondary link, a limitation is imposed by the 3G-GPRS mobile network coverage existing at the location of the system.

## 6. Results: Test Campaigns

In order to validate the correct functioning of the developed system, several unit tests and sea trials were made.

Unit tests have been carried out in the laboratory to validate data provided from the inertial Invensense MPU sensors (both accelerometers and gyroscopes), and errors were empirically characterized. Furthermore, GPS positioning data accuracy have been characterized, as shown in Section 3.5.

**Figure 19:**
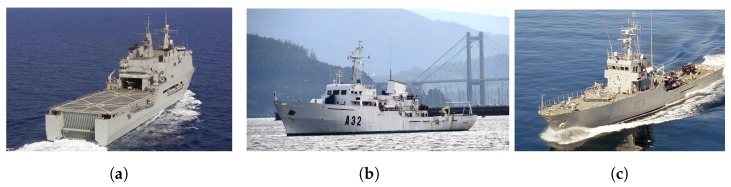
Measurement validation ships: (**a**) AAS“Castilla”; (**b**) HSS“Tofiño”; (**c**) PB“Tabarca”.

Sea trials were carried out to analyze and validate the attitude and position data of ships. Different campaigns were performed aboard three Spanish Navy vessels: Amphibious Assault Ship L-52 “Castilla” (Figure 19a), Hydrographic Survey Ship A-32 “Tofiño” (Figure 19b) and Patrol Boat P-28 “Tabarca” (Figure 19c).

Finally, the main and secondary radio links were also validated, demonstrating the correct functioning of the entire system.

### 6.1. Attitude Validation

Pitch, yaw and roll data of the three ships were recorded with the sensor nodes for long periods of time and in different locations on the ship.

The Tofiño vessel is equipped with a high performance inertial unit called the MRU (Motion Reference Unit), which was employed as a reference to contrast the attitude data measurements made by the nodes. This MRU, Model MRU-5 from Seatex [24], installed approximately at the center of gravity of the ship and used for bathymetric surveys, is composed by a three-axial accelerometer and a three-axial gyroscope, providing $0.02^{\circ }$ angle accuracy for both roll and pitch determination.

**Figure 20:**
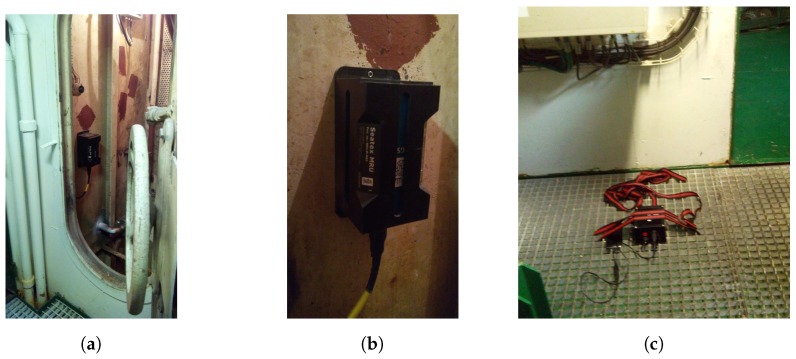
Installation for attitude data measurement validation on ship: (**a**) Motion Reference Unit (MRU) compartment; (**b**) MRU-5 from Seatex; (**c**) measurement node close to MRU.

For comparing measurement data, a node was installed (Figure 20c) near the MRU (Figure 20a,b) and aligned with it.

Roll angle determination from the low cost MPU-9250 data have shown a Mean Absolute Error (MAE) of $0.58^{\circ }$ and a Root Mean Square Error (RMSE) of $0.77^{\circ }$ in contrast to the data obtained from the MRU.

**Figure 21:**
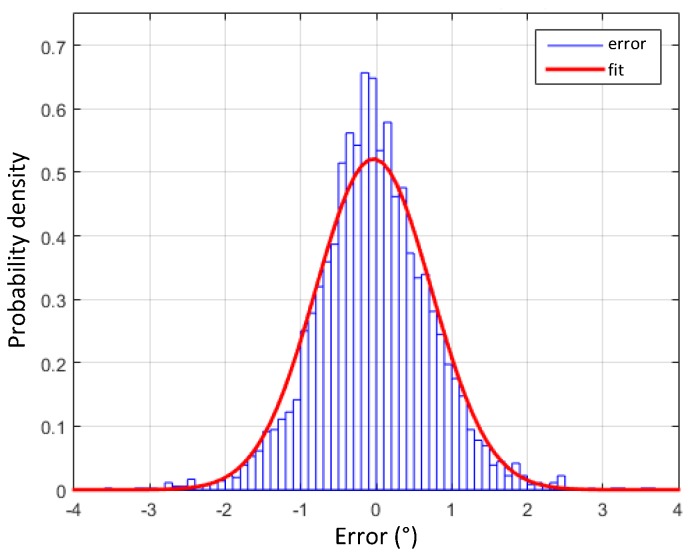
Probability density function of error in the roll angle estimation.

Figure 21 shows the measured roll error distribution obtained from the MPU-9250 IMU compared with the MRU, fitting a normal distribution with mean $\mu =-0.036^{\circ }$ and variance $\sigma^{2}=0.59^{\circ }$. The error in the roll angle estimation is always lower than $1.22^{\circ }$ in at least 95% of the cases.

When data from three redundant IMU are fused, MAE is lowered to $0.19^{\circ }$, and RMSE is improved to $0.69^{\circ }$, as shown in Table 3.

The error in the pitch angle determination has similar results, with a fitted measured pitch error normal distribution with mean $\mu =-0.284^{\circ }$ and variance $\sigma^{2}=1.79^{\circ }$. Table 4 reflects pitch angle error obtained when 1 to 4 IMUs are used.

### 6.2. Position and COG Validation

**Figure 22:**
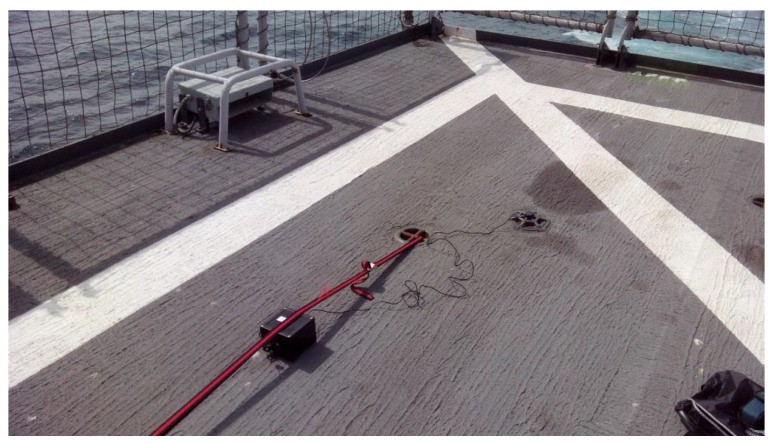
GPS position measurement over the helideck of the Castilla ship.

For testing the position data, GPS information was obtained from the onboard Castilla DGPS (Differential GPS) (Figure 22) and compared with the data provided by the GPS of the measurement node in various campaigns.

**Figure 23:**
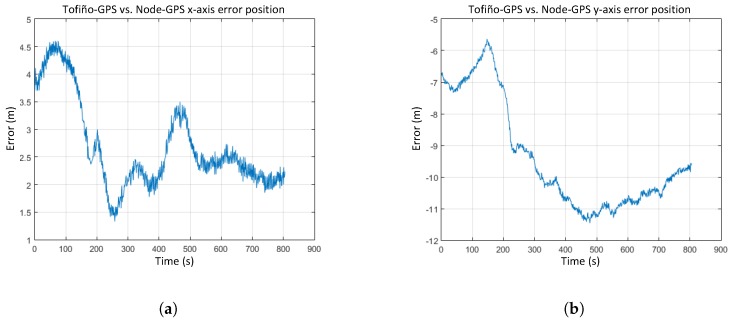
Error on position determination: (**a**) *x* axis. (**b**) *y* axis.

As shown in Figure 23, position errors in the *x* and *y* axes have an offset value because both GPS receivers were installed at different locations on the ship. RMS errors obtained in position determination are under 0.8 m in the *x* coordinate and 1.7 m in the *y* coordinate.

COG data supplied by the node were also compared with the those provided by the ship’s DGPS, leading to an error of $0.41^{\circ }$ RMS.

### 6.3. Radio Data Link Validation

**Figure 24:**
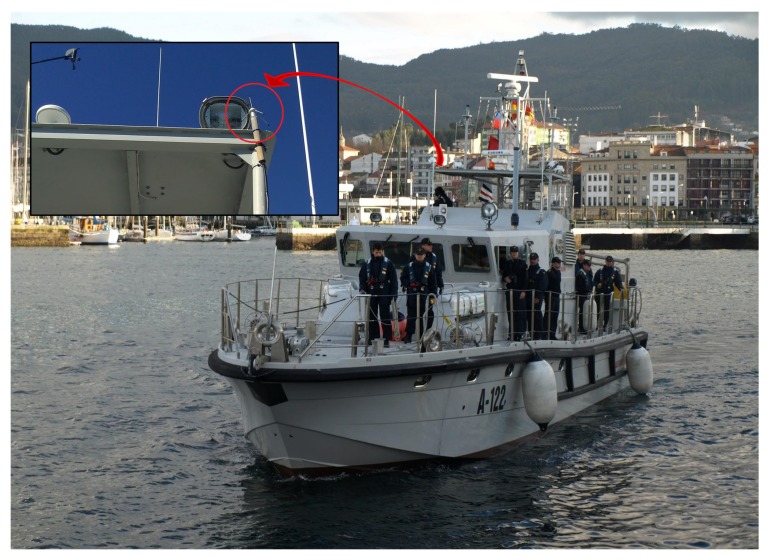
Instruction boat with the measurement node and the omnidirectional antenna for the radio link test.

For validating the main radio link, data transmission was done from a measurement node installed on an instruction boat (Figure 24). An omnidirectional monopole antenna was attached to the ship castle. The on-shore station is equipped with a directional Yagi antenna, as shown in Figure 4b, which is always pointed to the ship location.

**Figure 25:**
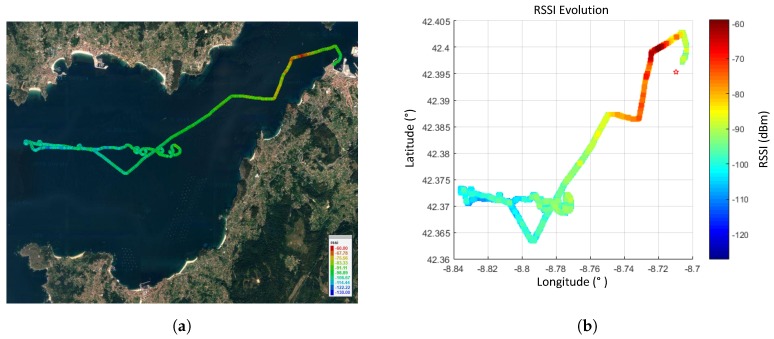
RSSI measurement trial: (**a**) Ría de Pontevedra scenario. (**b**) RSSI values obtained.

The RSSI (Received Signal Strength Indicator) value measured by the XBee module on the node was used to provide the received power values. According to the XBee module datasheet [11], RSSI values lower than -110 dBm must be discarded because they are under the sensitivity threshold of the receiver originating a loss of the radio link. Figure 25 shows an RSSI measurement scenario for a sea trial conducted at the Ría de Pontevedra.

**Figure 26:**
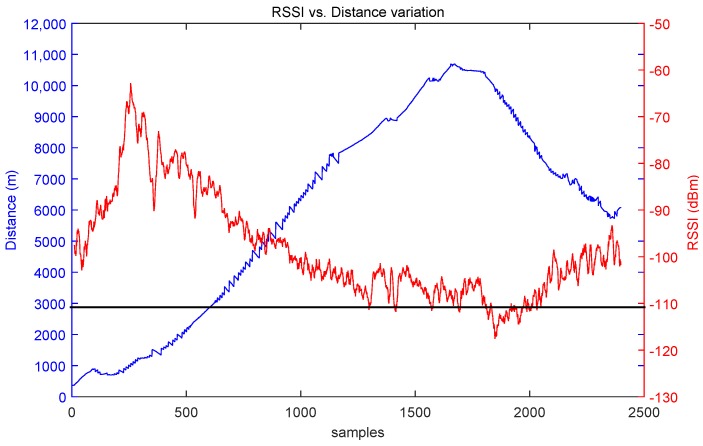
RSSI (Receiver Signal Strength Indicator) variation with the ship-to-shore distance.

Figure 26 shows the RSSI evolution as a function of the ship-to-shore range. The signal has been averaged to smooth the RSSI variations using a median filter of 10 samples. The first samples are not valid because they correspond to a signal blockage due to a nearby building. The sensitivity value is achieved at 8.5 km. Similar results were obtained after several measurements carried out at the same location, stating that approximately 8 km is the practical limit of the radio link.

## 7. Conclusions

The design, development and implementation of a low cost system for the measurement, recording and real-time transmission of attitude data (yaw, pitch and roll) and the position of ships at sea have been exposed. With this system, the high-resolution radar images of collaborating ships can be corrected. The system has been successfully tested in several Spanish Navy ships, as much in small boats as in large vessels. The validation has been obtained not only for the attitude-positioning measurements, but also for the radio data link.

The reliability of the system has been proven with a good XBee RF link stability and adequate coverage, though a 3G-GPRS radio link for extra dependability is provided. Moreover, the use of redundant inertial sensors improves the data availability and reduces the attitude angle estimation errors regarding the data provided by high performance equipment. It must be pointed out that the telemetry system is based on low cost MEMS inertial sensors and on the use of dual technologies and COST components; consequently, the performance observed during the validations must be satisfactorily assessed.

Autonomy of the measurement node and easiness of on-board installation are ensured. The inertial system does not need previous stabilization, and it only requires a quick calibration. Additionally, the open and flexible platform (software as much as hardware) is adequate for experimentation, as it uses open interfaces and standard buses that allow easily adding new hardware and sensors. Eventual reprogramming of the nodes is possible thanks to the implemented OTAP feature that allows modifying procedures on the fly and remotely changing the operating parameters of the system.

## Figures and Tables

**Table 1:** Main characteristics of the inertial sensor MPU-9250.

Accelerometer	
Range	±2, ±4, ±8 and ±16 g
Resolution	0.00060 m/s${}^{2}$ to 0.00479 m/s${}^{2}$
Initial Zero Bias	X,Y:60 mg; Z:80 mg
Initial Scale Factor Variation	3%
Nonlinearity	0.5%
**Gyroscope**	
Range	±250, ±500, ±1000 and ±2000${}^{\circ }/$s
Resolution	0.00763 to 0.06098${}^{\circ }/$s
Initial Zero Rate	±5${}^{\circ }/$s
Initial Scale Factor Variation	3%
Nonlinearity	0.1%
**Magnetometer**	
Range	±4800 μT
Resolution	0.6 μT/LSB (14 bit) or 15 μT/LSB (16 bit)
Initial Scale Factor Variation	5%

**Table 2:** Energy consumption of the measurement node.

Node Consumptions	
Main board	15 mA
GPS module ${}^{1}$	32 mA
XBee module ${}^{2}$	500 mA
IMU block ${}^{3}$	10 mA
**Total**	557 mA

${}^{1}$ Jupiter-JN3 from Telit [23]; ${}^{2}$ XBee-PRO-868 from Digi [11]; ${}^{3}$ three MPU-9250 from Invensense @ 3.5 mA each [13].

**Table 3:** Roll angle error improvement obtained from multiple inertial sensors.

Roll Angle Error		
**No. of IMUs**	$\sigma_{e}^{2}$	**e** ${}_{95\%}$
1	$0.585^{\circ }$	<1.22${}^{\circ }$
2	$0.292^{\circ }$	<0.85${}^{\circ }$
**3**	**0.195**${}^{\circ }$	<**0.69**${}^{\circ }$
4	$0.146^{\circ }$	<0.59${}^{\circ }$

**Table 4:** Pitch angle error improvement obtained from multiple inertial sensors.

Pitch Angle Error		
**No. of IMUs**	$\sigma_{e}^{2}$	**e**${}_{95\%}$
1	$0.179^{\circ }$	<0.98${}^{\circ }$
2	$0.089^{\circ }$	<0.77${}^{\circ }$
**3**	**0.059**${}^{\circ }$	<**0.68**${}^{\circ }$
4	$0.044^{\circ }$	<0.60${}^{\circ }$

## Abbreviations

The following abbreviations are used in this manuscript:

CEP	Circular Error Probable
COG	Course Over Ground
COTS	Commercial Off-The-Shelf
DGPS	Differential Global Positioning System
GPS	Global Positioning System
HRR	High Resolution Radar
IMU	Inertial Measurement Unit
ISAR	Inverse Synthetic Aperture Radar
ISM	Industrial, Scientific and Medical
MAE	Mean Absolute Error
MEMS	Micro Electro-Mechanical Systems
MRU	Motion Reference Unit
OTAP	Over The Air Programming
RCS	Radar Cross-Section
RF	Radio Frequency
RMSE	Root Mean Square Error
RSSI	Receiver Signal Strength Indicator
RTC	Real-Time Clock
UART	Universal Asynchronous Receiver Transmitter
WSN	Wireless Sensor Networks
